# Familial Longevity is Associated with an Attenuated Thyroidal Response to Recombinant Human Thyroid Stimulating Hormone

**DOI:** 10.1210/clinem/dgaa195

**Published:** 2020-04-18

**Authors:** Ana Zutinic, Hanno Pijl, Bart E Ballieux, Ferdinand Roelfsema, Rudi G J Westendorp, Gerard J Blauw, Diana van Heemst

**Affiliations:** 1 Department of Internal Medicine, Divis ion of Gerontology and Geriatrics, Leiden University Medical Centre, ZA, Leiden, The Netherlands; 2 Department of Internal Medicine, Division of Endocrinology and Metabolic Diseases, Leiden University Medical Centre, ZA, Leiden, The Netherlands; 3 Department of Clinical Chemistry and Laboratory Medicine, Leiden University Medical Centre, ZA, Leiden, The Netherlands; 4 Public Health and Center for Healthy Aging, University of Copenhagen, Copenhagen, Denmark

**Keywords:** Thyroid, longevity, recombinant human TSH, responsivity

## Abstract

**Context:**

Longevity is associated with higher circulating levels of TSH in the absence of differences in circulating thyroid hormones (TH), as previously observed in F2 members of long-lived families (F2-LLS) and their partners (F2-Con). The mechanism underlying this observed difference remains unknown.

**Objective:**

We hypothesized that the thyroid gland of members from long-lived families are less responsive to TSH stimulation, thereby requiring higher circulating TSH levels to maintain adequate TH levels.

**Methods:**

We performed a case-control intervention study with a single intramuscular (gluteal) injection with 0.1 mg recombinant human TSH in a subgroup of 14 F2-LLS and 15 similarly aged F2-Con. They were followed for 4 days. No serious adverse events were reported. For analyses, we compared time trajectories of TSH and TH, and the ratio of TH to TSH using area under the curve (AUC) calculations.

**Results:**

The AUC free T4/AUC TSH ratio was significantly lower in F2-LLS than in F2-Con (estimated mean [95% confidence interval] 1.6 [1.2-1.9] and 2.2 [1.9-2.6], respectively, *P* = 0.01). The AUC thyroglobulin/AUC TSH ratio was also lower in F2-LLS than in F2-Con (median [interquartile range] 2.1 [1.4-3.6] and 3.2 [2.7-7.4], respectively, *P* = 0.04). We observed the same trend with the AUC free T3/AUC TSH ratio, although the difference was not statistically significant (estimated mean [95% confidence interval] 0.6 [0.4-0.7] and 0.7 [0.6-0.8], respectively, *P* = 0.07).

**Conclusions:**

The present findings show that members of long-living families have a lower thyroid responsivity to TSH compared with their partners.

Thyroid status changes with age (1) and plays an important role in multiple physiological processes. Circulating levels of thyroid hormones (THs) are tightly regulated through an interplay of feedforward and feedback mechanisms. Hypothalamic TRH stimulates the secretion of TSH from the pituitary that stimulates the thyroid gland to produce and release THs into the circulation. Increases in circulating levels of THs are centrally monitored and lead to inhibition of the release of TRH and TSH, which puts a halt to further increases in TH production. With aging, several changes occur in circulating parameters of the thyroid axis. Most strikingly, TSH levels tend to increase with age, a trend that was observed to extend into advanced ages (2), and that might be explained by selective survival of people with a genetic or familial predisposition for relatively higher TSH (3, 4).

In line with these earlier findings, we observed that in advanced middle age, in a substudy from the Dutch Leiden Longevity Study (LLS) comprising 38 participants from whom blood samples were taken every 10 minutes during 24 hours, that members from long-lived families (F2-LLS) had on average an 0.8 mU/L higher serum concentration of TSH than the similarly aged reference group (their partners, F2-Con), whereas TH levels were comparable between groups (5).

Additionally, in the same cohort, we observed a stronger temporal relationship between TSH and free T3 in F2-LLS than in F2-Con, but no differences in the feedback and forward interplay between TSH and THs (6). The bioactivity of TSH has been shown to not differ between F2-LLS and F2-Con (5).

The aim of the present study is to test the hypothesis that the thyroid gland of F2-LLS is more resistant to stimulation with TSH compared with the thyroid gland of their partners, F2-Con. To investigate this, we recruited F2-LLS and F2-Con from a subgroup of LLS for a challenge study with a single dose of recombinant human TSH (rhTSH, Thyrogen, Genzyme Corp., Framingham, MA), where we hypothesized that upon administration of the same low dose of recombinant human TSH, F2-LLS will have a lower thyroidal response than F2-Con.

## Materials and Methods

### Study population

The LLS was founded in 2002 and designed to investigate genotypes and phenotypes underlying interindividual differences in familial longevity in humans (7). In the LLS, family members of 2 different generations were included, comprising an F1 generation of long‐lived siblings from 421 Caucasian long-living families (men aged 89 and older, women aged 91 and older) living in The Netherlands in early 2000s, without any restrictions on health or demographics (8). The offspring of these long-lived F1 siblings were also asked to participate in the study, with the offspring’s partners as controls, thereby creating a case group enriched for longevity (F2-LLS) and a control group with similar lifestyle factors and socio-economic status, but without selection for familial predisposition to longevity (F2-Con).

Subjects were recruited for the TSH challenge study from the subgroup of LLS previously studied in terms of thyroidal status between F2-LLS and F2-Con (5), and excluded based on the following exclusion criteria. The exclusion criteria were: laboratory results (hemoglobin < 7.1 mmol/L, TSH > 4.0 mU/L, free T4 (fT4) < 9 pmol/L or > 24 pmol/L, thyroid peroxidase antibody positivity (> 35 kU/L)), medical history (cardiac arrhythmias, [history of] thyroid diseases, renal, hepatic, or endocrine disease, or any other significant chronic disease), medication use (hormone therapy, thyroid medication), lifestyle factors (nicotine abuse, [history of] alcohol abuse [> 28 units per week]), and other factors (difficulty inserting an intravenous cannula, participation in other research projects within the past 3 months, participation in 2 or more projects in 1 year, evaluation by a physician as too frail, or vulnerable to participate).

### Clinical protocol

Participants were admitted into the study after passing medical screening. The TSH challenge study consisted of 4 consecutive study days at Leiden University Medical Centre. On the morning of study day 1, an IV cannula was placed in a forearm vein, blood was withdrawn at baseline and rhTSH was administered through IM injection (0.1 mg/mL in 1 mL, gluteal muscle). The time of injection was used as reference, time zero. Blood was sampled at a high frequency following injection for optimal detection of circulating parameters reflecting the thyroidal response to rhTSH. In the first hour after injection, blood was sampled every 15 minutes. Between 1 and 3 hours after injection, blood was sampled every 30 minutes, and finally between 3 and 8 hours after injection, every hour. During study day 1, subjects received 2 standardized meals (2 hours and 5 hours after rhTSH injection), each consisting of 600 kcal (2 × 125mL Nutridrink Compact, Nutricia Advanced Medical Nutrition, Zoetermeer, The Netherlands). On study day 2, 3, and 4, additional blood samples were obtained at respectively 24, 48, and 72 hours after rhTSH injection. Outside of these times, subjects were at their leisure.

The blood samples obtained at baseline, 15 minutes through 2 hours, and 24, 48, and 72 hours after injection were drawn when participants were in the fasted state.

In total, 255.5 mL of blood was withdrawn from each subject across 17 time points (14 on study day 1, and 1 each on days 2, 3, and 4).

Height, weight, and body composition were measured on study day 2. Body composition was measured with a Bioelectrical Impedance Analysis meter at a fixed frequency of 50 kHz (Bodystat 1500 Ltd, Isle of Man, British Isles (9)).

The study was designed in accordance with the declaration of Helsinki and has been approved by the Medical Ethical Committee of the Leiden University Medical Centre. It is registered at Leiden University Medical Centre under the protocol P16.107 and with EudraCT under the number 2016-001497-15. All subjects gave written informed consent before the screening visit.

### Handling of samples

Serum samples were kept at room temperature for 60 minutes to clot before processing at the Department of Clinical Chemistry and Laboratory Medicine, Leiden University Medical Centre, The Netherlands. Samples were centrifuged for 10 minutes at 2350*g* relative centrifugal force at a temperature of 20°C. After being transferred to 500 microliter aliquots, serum samples were stored at –20°C before permanent storage at –80°C until analysis.

### Laboratory measurements

Laboratory measurements in serum samples were performed after all subjects had completed the study. Samples from 6 participants were measured as a pilot, followed by measurements in the remaining 23 participants’ samples. All measurements were performed with the same lot number. For each participant, samples from the different time points were measured in the same batch.

### Assays and assay performance

All measurements were performed with fully automated, software monitored equipment and diagnostics from Roche Diagnostics (Almere, The Netherlands) at the Department of Clinical Chemistry and Laboratory Medicine at Leiden University Medical Centre, The Netherlands. Aspartate aminotransferase (catalog no. 11876848216), alanine aminotransferase (catalog no. 11876805216), and creatine (catalog no. 5168589190) for estimating glomerular filtration rate (GFR) were measured from a fasted morning serum sample using the Modular P800 clinical chemistry analyser. GFR was calculated using the CKD-EPI calculation. Thyroid parameters TSH (catalog no. 11731459122, research reference identifier [RRID]: AB_2756377), fT4 (catalog no. 6437281190, RRID: AB_2801661), T4 (catalog no. 12017709122, RRID: AB_2756378), fT3 (catalog no. 6437206190, RRID: AB_2827368), and T3 (catalog no. 11731360122, RRID: AB_2827369) were measured in serum by an immunoassay using Roche cobas8000 with an E602 module. The coefficients of variation were 2.36 (SD 0.52) for TSH, 5.55 (SD 2.28) for fT4, 2.06 (SD 0.58) for fT3, 5.25 (SD 0.34) for T3, and 2.88 (SD 0.41) for thyroglobulin (Tg).

### Statistical analyses

Descriptive statistics were used to summarize group characteristics. Independent samples *t* test, Mann-Whitney *U* test, and χ ^2^ test were used, depending on the characteristics of the variable (normally distributed, not normally distributed, and categorical, respectively), to statistically test for differences between (male and female) F2-LLS and F2-Con regarding demographics, anthropometrics, and laboratory measurements. We used log transformation to normally distribute data that were not normally distributed, or nonparametric testing in the case of data that could not be transformed to normal distribution. The cumulative area under the curve (AUC) was calculated using a trapezoid model with Matlab (Mathworks, Natick, MA). Here, we used only the 72-hour time point and thus the total integrated area. General linear modelling was used to investigate differences in TSH and TH kinetics between F2-LLS and F2-Con. To answer our research question on whether the thyroid gland of F2-LLS is less responsive to rhTSH stimulation than those of F2-Con, we calculated the ratio of total circulating THs (AUC fT4, AUC fT3, and AUC Tg) to total circulating TSH during the study (AUC TSH). Pearson correlation was used to test the correlation between GFR and AUC TSH. Linear mixed modelling was used to test for differences in AUC ratios between F2-LLS and F2-Con adjusted for age and gender. In all analyses, *P* ≤ 0.05 was considered statistically significant.

We used power calculations to determine group size. A 2-sided significance level of 5% was used and the power was set at 80%. A sample size of 10 participants will have 80% power to measure a 2.71 pmol/L difference in fT4 levels after rhTSH stimulation. In addition, including 10 participants per group will have 92% power to detect a 0.73 pmol/L difference in fT3 levels after rhTSH stimulation. To overcome possible dropout resulting from difficulties with blood sampling or the laboratory measurements 15 participants were included in each group.

Programs used for statistical analyses were SPPS for Windows, version 23 (SPSS, Chicago, IL) and Matlab (The MathWorks Inc, Natick, MA). Graphs were made using Microsoft Office Excel 2016 and GraphPad Prism for Windows, version 8.1.1 (330) (GraphPad Software, Inc, San Diego, CA).

## Results

### Inclusions

**Figure 1.**
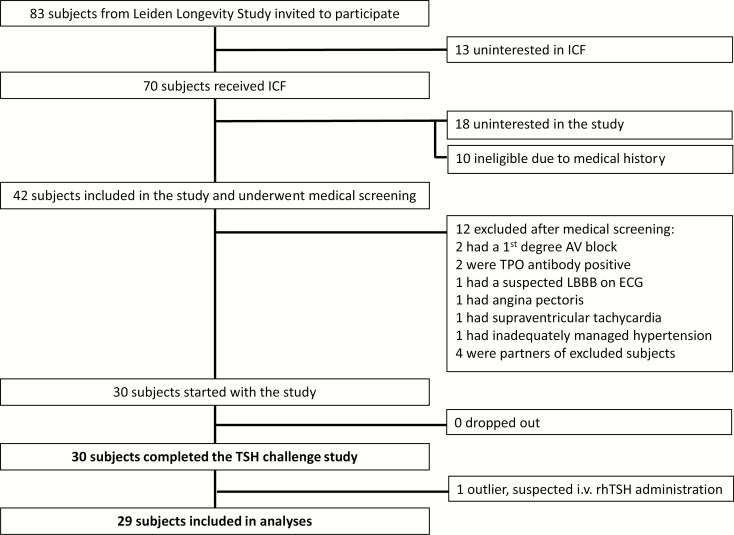
Recruitment and inclusion flowchart of subjects for the TSH challenge study. AV, atrioventricular; ECG, electrocardiogram; ICF, informed consent form; LBBB, left bundle branch block; TPO, thyroid peroxidase.

The recruitment and inclusion flow chart of subjects for the study is presented in Fig. 1. In total, 83 individuals were selected and invited by telephone to participate. Thirteen individuals were not interested in receiving the informed consent form. Of 70 individuals who did receive an informed consent form, 18 were not interested in participating in the study and 10 had significant medical history that made them unsuitable candidates. Forty-twi individuals were included in the study and have undergone a medical screening. Twelve individuals were excluded on the basis of the findings from the medical screening. Consequently, 30 subjects were included and have completed the study. The F2-LLS selected were nonconsanguineous. One participant was excluded from analyses because of suspected IV rhTSH administration based on TSH peak and concentration profile during the study. In this subject, the TSH peak of 243 mU/L (> 4 SD from mean of other F2-LLS) was reached 15 minutes following 0.1 mg rhTSH administration (versus on average 7 hours in other F2-LLS). The TSH concentration subsequently remained > 4 SD from the mean of other F2-LLS throughout study day 1, although subsequently decreasing and eventually reaching levels below baseline by study day 4.

### Group characteristics

Baseline characteristics of the study population are presented in Table 1. The F2-LLS (n = 14) and F2-Con (n = 15) were similar regarding age, sex, and body mass index. They comprised a healthy, high middle-age population. Parental age was higher in F2-LLS than in F2-Con (*P* < 0.01 for mothers and *P* = 0.02 for fathers), confirming the longevity phenotype on which F2-LLS were selected for the Leiden Longevity Study. Both groups had a mean kidney function within normal range (GFR > 60 mL/min per 1.73 m^2^), although kidney function was slightly lower in F2-LLS than in F2-Con (*P* = 0.04). One participant, a female F2-LLS, had GFR 57 at screening, but was included in the study because of absence of any indication of chronic (kidney) disease. GFR was not significantly correlated with AUC TSH (Pearson correlation *r* = -0.28, *P* = 0.15). Baseline TSH was significantly higher in F2-LLS than in F2-Con (*P* = 0.04), whereas other thyroid hormones were similar between F2-LLS and F2-Con (fT4 *P* = 0.12, fT3 *P* = 0.15, Tg *P* = 0.18), confirming the selection of participants with the longevity-associated TSH phenotype.

**Table 1.** Baseline Characteristics of the Study Population

	F2-LLS (n = 14)	F2-Con (n = 15)
**Demographics**		
Age mother, years^a^	93 (91-97)	75 (69-85)
Age father, years^a^	93 (73-96)	78 (61-82)
Male, n (%)	8 (57)	6 (40)
Age, years	69 (5)	69 (6)
**Anthropometrics**		
BMI, kg/m^2^	25.8 (4.3)	26.3 (4.4)
Weight, kg	78.1 (15.4)	78.1 (15.0)
Height, cm	173.7 (10.9)	171.9 (9.0)
Fat mass, kg	23.9 (7.2)	26.7 (8.4)
Lean mass, kg	54.1 (12.9)	50.7 (13.2)
**Laboratory measurements**		
GFR, mL/min per 1.73 m^2^	71.2 (13.9)	80.3 (8.4)
AST, U/L	22.3 (4.1)	24.6 (7.1)
ALT, U/L	19.6 (5.3)	22.1 (8.3)
Baseline TSH, mU/L	3.3 (1.7)	2.2 (1.0)
Baseline fT4, pmol/L^a^	13.9 (13.0-15.8)	15.3 (14.3-15.7)
Baseline fT3, pmol/L	4.6 (0.5)	4.3 (0.5)
Baseline Tg, µg/L^a^	10.7 (6.9-22.9)	14.3 (10.2-33.6)

All values are mean (standard deviation) unless otherwise stated.

^a^Median (interquartile range).

Abbreviations: ALT, alanine transaminase; AST, aspartate transaminase; BMI, body mass index; F2-Con, partners of F2-LLS; F2-LLS, members of long-living families; fT3, free T3; fT4, free T4; GFR, glomerular filtration rate; Tg, thyroglobulin.

### Thyroid response to rhTSH challenge

**Figure 2.**
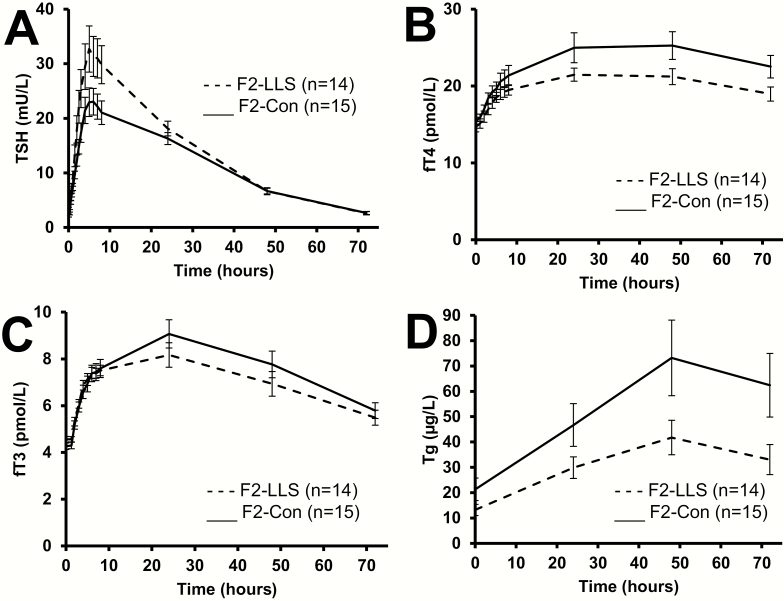
Four-day (72 hours) profile of mean circulating TSH and mean circulating thyroid hormones in members of long-lived families, F2-LLS (n = 14), and their partners, F2-Con (n = 15) following injection with 0.1 mg recombinant human TSH. (A) Mean circulating TSH, general linear model during first study day: *P* value = 0.031 between offspring and partners, within time *P* < 0.0001, offspring or partner over time *P* = 0.029, (B) mean circulating free T4 (fT4), (C) mean circulating free T3 (fT3), and (D) mean circulating thyroglobulin (Tg). Black lines: F2-Con; dashed lines: F2-LLS. Error bars: standard error of the mean.

**Figure 3.**
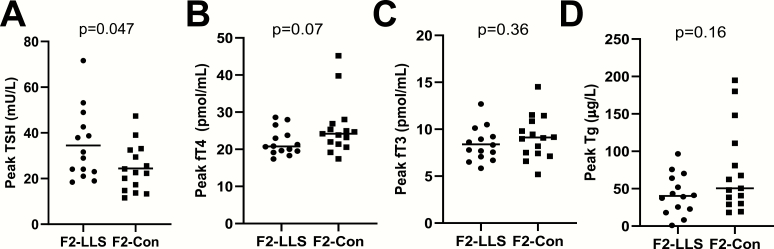
Peak values of circulating TSH and circulating thyroid hormones in members of long lived-families, F2-LLS (n = 14) and their partners, F2-Con (n = 15) following injection with 0.1 mg recombinant human TSH. (A) Peak values of TSH, horizontal line represents the mean, **B)** peak values of free T4 (fT4), horizontal line represents the median, (C) peak values of free T3 (fT3), horizontal line represents the mean, and (D) peak values of thyroglobulin (Tg), horizontal line represents the median. *P* value < 0.05 was considered statistically significant.

Following injection with 0.1 mg rhTSH, circulating TSH levels increased to supraphysiologic levels in F2-LLS and F2-Con throughout study days 1, 2, and 3, and returned to baseline by day 4 (mean [SEM] in F2-LLS 2.6 [0.3] mU/L and in F2-Con 2.7 [0.3] mU/L), as shown in Fig. 2A. The mean (SEM) peak TSH value was higher in F2-LLS than in F2-Con (34.5 [4.1] mU/L and 24.5 [2.7] mU/L, respectively; *P* = 0.047), as shown in Fig. 3A. Both peak values were reached on average 7 hours after injection (*P* = 0.87 between F2-LLS and F2-Con). Generalized linear model calculations show that circulating TSH was different between F2-LLS and F2-Con during the first 8 hours following rhTSH administration, *P* = 0.031, as well as different in time progression, *P* < 0.0001. However, AUC calculations show that mean (SEM) AUC TSH for the whole study (72 hours) was not significantly different between F2-LLS and F2-Con (985 [76] mU/L and 824 [57] mU/L, respectively, *P* = 0.10).

Following 0.1 mg rhTSH injection, THs increased in both F2-LLS and F2-Con (Fig. 2B-D), with most participants reaching peak values of fT4 and fT3 24 hours after injection (interquartile range 24-48 and 8-24 hours, respectively) and peak values of Tg 48 hours after injection (interquartile range 48-48 hours). Peak values of TH were similar in F2-LLS and in F2-Con (Fig. 3B–D). Median (IQR) peak fT4 was 20.8 (19.4-24.5) pmol/L in F2-LLS and 24.2 (21.4-26.9) pmol/L in F2-Con, *P* = 0.07. Mean (SEM) peak fT3 was 8.4 (0.5) pmol/L in F2-LLS and 9.1 (0.6) pmol/L in F2-Con, *P* = 00.36. Median (IQR) peak Tg was 40.3 (22.7-64.0) µg/L in F2-LLS and 50.5 (30.2-110.7) µg/L in F2-Con, *P* = 00.16. The whole study median (IQR) AUC for fT4 was 1411 (1276-1630) pmol/mL in F2-LLS and 1619 (1404-1713) pmol/mL in F2-Con, *P* = 00.10. The whole study AUC fT3 was not significantly different between F2-LLS and F2-Con (mean (SEM) 506 [29] pmol/mL and 548 (34) pmol/mL in F2-Con, respectively, *P* = 00.36). The whole study AUC Tg was not significantly different between F2-LLS and F2-Con (median [IQR] 2277 [1302-3126] µg/L and 2977 [1804-6512] µg/L, respectively, *P* = 00.18.

**Figure 4.**
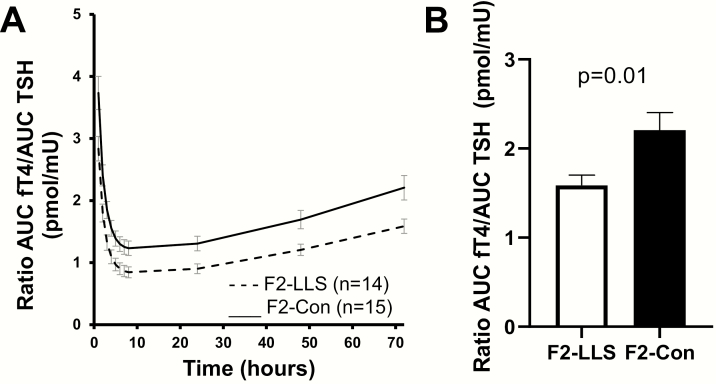
The ratio of circulating free T4 (fT4) to circulating TSH in members of long-lived families, F2-LLS (n = 14) and their partners, F2-Con (n = 15), based on area under the curve calculations (AUC), following injection with 0.1 mg recombinant human TSH. (A) Four-day profile of the mean AUC ratio of circulating fT4 to circulating TSH. General linear modelling between offspring and partners *P* value = 0.04. (B) Whole study (72 hours) mean area under the curve ratio of circulating fT4 to circulating TSH. Error bars represent standard error of the mean.

To investigate the response of the thyroid gland to TSH, we calculated the AUC fT4/AUC TSH ratio. The AUC fT4/AUC TSH ratio was lower in F2-LLS than in F2-Con at all time points, including baseline, as shown in Fig. 4A (generalized linear model *P* = 00.04 between F2-LLS and F2-Con). The whole study (72 hours) AUC fT4/AUC TSH ratio was significantly lower in F2-LLS than in F2-Con (mean [SEM] 1.6 [0.1] pmol/mU and 2.2 [0.2] pmol/mU, respectively, *P* = 00.01), as shown in Fig. 4B. When adjusted for age and gender, the AUC fT4/AUC TSH ratio remained significantly different between F2-LLS and F2-Con (estimated mean [95% confidence interval] 1.6 [1.2-1.9] pmol/mU and 2.2 [1.9-2.6] pmol/mU, respectively, *P* = 00.01).

**Figure 5.**
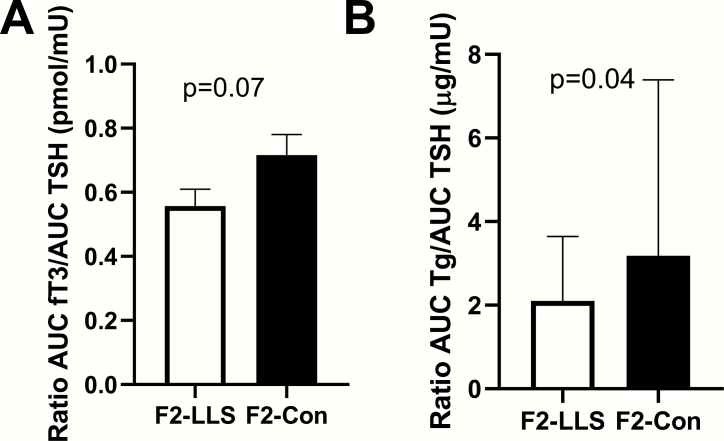
The ratio of circulating thyroid hormones free T3 (fT3) and thyroglobulin (Tg) to circulating TSH in members of long-lived families, F2-LLS (n = 14) and their partners, F2-Con (n = 15), based on whole study (72 hours) area under the curve calculations (AUC), following injection with 0.1 mg recombinant human TSH. (A) Mean AUC ratio of circulating fT3 to circulating TSH in offspring and partners, *P* = 0.07, and (B) median area under the curve ratio of circulating Tg to circulating TSH in offspring and partners, *P* = 0.04. *P* value < 0.05 was considered statistically significant. Error bars represent standard error of the mean in panel A, and interquartile range in panel B.

We investigated the relationship of secondary output parameters of the thyroid gland (fT3 and Tg) to TSH. We again observed the trend of a lower AUC fT3/AUC TSH ratio in F2-LLS than in F2-Con (Fig. 5A), although the difference was not statistically significant (mean [SEM] 0.6 [0.1] pmol/mU and 0.7 [0.1] pmol/mU, respectively, *P* = 00.07). When adjusted for age and gender, the difference in AUC fT3/AUC TSH ratio between F2-LLS and F2-Con remained not significant (estimated mean [95% confidence interval] 0.6 [0.4–0.7] pmol/mU and 0.7 [0.6–0.8] pmol/mU, respectively, *P* = 00.07). The AUC Tg/AUC TSH (Fig. 5B) was lower in F2-LLS than in F2-Con (median [IQR] 2.1 [1.4-3.6] µg/mU and 3.2 [2.7-7.4] µg/mU, respectively, *P* = 00.04). When adjusted for age and gender, the AUC Tg/AUC TSH ratio remained significantly different between F2-LLS and F2-Con (estimated mean [95% confidence interval] 2.6 [1.1-4.0] µg/mU and 4.8 [3.3-6.2] µg/mU, respectively, *P* = 00.04).

## Discussion

In this study, we investigated whether human familial longevity is associated with lower thyroidal responsivity to stimulation by TSH. Overall, we demonstrated that administration of 0.1 mg rhTSH results in lower fT4 to TSH ratio in members from long-living families compared with their partners, thereby supporting our hypothesis that longevity is associated with lower thyroidal responsivity to TSH.

Whereas both F2-LLS and F2-Con had circulating fT4, fT3, and Tg levels within normal range throughout the study, F2-LLS had a lower AUC fT4/AUC TSH than F2-Con. This was the case at baseline, but also under challenge conditions, indicating the perseverance of lower thyroid responsivity in F2-LLS compared with F2-Con even in the presence of supraphysiologic circulating TSH levels. Secondary parameters of the thyroid gland, namely fT3 and Tg, showed a similar trend, with the AUC fT3/AUC TSH nonsignificantly lower and AUC Tg/AUC TSH significantly lower in F2-LLS compared with F2-Con.

TSH and TH profiles upon administration of 0.1 mg rhTSH in our study were comparable to those from previous studies concerning the use of rhTSH in healthy young and middle-aged subjects (10-14). The dose of 0.1 mg rhTSH was adequate to increase circulating TSH to supraphysiologic levels in young and middle-aged subjects, where levels of circulating TSH increased 2 hours after intramuscular injection with rhTSH and return to baseline over the course of 3 to 4 days (10, 11, 14), corresponding to TSH concentration profiles in our study. Peak TSH values following injection were variable in healthy young and middle-aged adults (14-16), as was also the case in our study. Despite reached supraphysiologic levels of circulating TSH, there were no serious adverse events or suspected unexpected serious adverse reactions in our study, indicating the safety of this dose in healthy older individuals, which could at least in part be due to sustained circulating TH within the normal range, as previously also reported (10, 14). Upon administration of the same dose of rhTSH, TSH concentrations over the first 8 hours (including peak TSH concentration) were higher in F2-LLS than in F2-Con. We have not found an explanation for this result and its implications remain unclear. Importantly, it has previously been reported that TSH concentrations following injection with rhTSH vary widely between individuals, and that these differences may be influenced by gender, age, and body composition (14, 15). In our study, age, body mass index, and gender distribution were comparable between the groups of F2-LLS and F2-Con. In addition, we did not find a significant difference in TSH peak concentrations after stimulation with rhTSH between men and women.

This study for the first time provides a mechanistic underpinning for the previously observed higher circulating TSH but similar TH levels in members of long-lived families compared with controls (5). Although the combination of higher TSH with similar levels of TH has not yet been studied in animal models of longevity, previous findings have reported negative associations between TH levels and lifespan in multiple animal models (1, 17). Interestingly, long-lived Ames and Snell mutant dwarf mice (18, 19), which exhibit a combined hormonal deficiency for GH, TSH, and prolactin show traits that are related to thyroid hormone deficiency, and supplementation of thyroid hormone during adulthood partly reduces their increased lifespan (20).

The study has several strengths. First, the high frequency of blood sampling following administration of 0.1 mg rhTSH allowed for observation in great detail of TSH pharmacokinetics during the first 8 hours after administration. Second, careful planning of laboratory measurements allowed for minimal intermeasurement variation, all samples were measured once all participants have completed the study, and the potential confounding effect of laboratory batch variation was avoided by using the same reagent and the same batch for all samples per participant. Finally, to study the mechanism underlying altered thyroid phenotype in familial longevity, we have selected participants from the pool of F2-LLS from long-living families and their partners in whom this difference in TSH phenotype has previously been found (5), thereby investigating this specific mechanism in the specific target population, which is a major strength of the study. Additionally, our study had strict health criteria to minimize any risk of adverse events and side effects in our high middle-age population under challenge conditions. This means we have selected a relatively healthy group of older individuals, with minimal confounding by comorbidities or polypharmacy, allowing us to optimally study the physiological effect of rhTSH on these subjects. Although the sample size was small, it was adequate to detect differences in fT4 levels between F2-LLS and F2-Con following 0.1 mg rhTSH administration, while remaining ethically responsible because of the high burden of the study for the participants.

The study has a couple of limitations. First, to minimize recruitment bottlenecks, we did not include a placebo control group to observe thyroid parameters throughout the study period without intervention. Hence, we cannot adjust for baseline thyroid parameters throughout the study. It is possible that the difference we have found between F2-LLS and F2-Con is therefore underestimated and would be even greater when adjusted for physiological thyroid values. Second, although conditions during study day 1 were standardized (fixed meal times, standardized meals, only water or tea for beverages), there were no alcohol abstinence guidelines during the remainder of the study. Because rhTSH is at least partly metabolized by the liver, it is possible that alcohol consumption during the remainder of the study has influenced TSH pharmacokinetics in some subjects. However, because most of the population consumed 1 to 2 glasses of alcohol per day in general and subjects consuming > 4 glasses of alcohol per day were not included in the study, the effect of alcohol on TSH kinetics is probably minimal. In future clinical studies with drugs metabolized by the liver, we recommend advising alcohol abstinence during the study or documenting alcohol consumption.

The principal finding of this study is that familial longevity is associated with lower thyroid responsivity to TSH. In future studies, we aim to investigate the influence of this finding on secondary tissues influenced by the thyroid axis, by measuring parameters of bone turnover, and different immune parameters to investigate possible extrathyroidal effects of TSH because it has been speculated that TSH has direct effects on tissues other than the thyroid gland. This hypothesis is supported by the observation that the TSH receptor is expressed in cells from several other tissues than the thyroid gland, including bone, adipose tissue, brain, and thymus (21-25). Moreover, other mechanisms underlying elevated TSH levels in the absence of elevated THs in F2-LLS from long-living families are possible. TSH bioactivity in these subjects has been tested and has been found to be similar between F2-LLS and F2-Con (5), but higher TH turnover in F2-LLS compared with F2-Con has not yet been investigated. Further studies, possibly through administration of TH in members of long-living families and their partners, are needed to investigate this.

In summary, familial longevity is associated with attenuated thyroid responsivity to an rhTSH challenge as observed by lower AUC fT4/AUC TSH and AUC Tg/AUC TSH ratio in members from long-lived families compared with controls. Further studies investigating extrathyroidal effects of TSH as well as turnover of TH in familial longevity are necessary to advance our understanding of the role of the thyroid axis in longevity.

## Abbreviations

AUC: area under the curve
F2-Con: partners of F2 members of long-lived families
F2-LLS: F2 members of long-lived families
fT4: free T4
GFR: glomerular filtration rate
LLS: Dutch Leiden Longevity Study
rhTSH: recombinant human TSH
TH: thyroid hormone
Tg: thyroglobulin.

## Additional Information

***Disclosure Summary:*** The authors have nothing to disclose.

***Data Availability:*** The datasets generated during and analyzed during the current study are not publicly available but are available from the corresponding author on reasonable request.
